# Ellagic Acid‐Loaded sEVs Encapsulated in GelMA Hydrogel Accelerate Diabetic Wound Healing by Activating EGFR on Skin Repair Cells

**DOI:** 10.1111/cpr.70064

**Published:** 2025-05-19

**Authors:** Lige Tian, Zihao Wang, Shengqiu Chen, Kailu Guo, Yaying Hao, Liqian Ma, Kui Ma, Junli Chen, Xi Liu, Linlin Li, Xiaobing Fu, Cuiping Zhang

**Affiliations:** ^1^ College of Graduate, Tianjin Medical University Tianjin China; ^2^ Medical Innovation Research Department PLA General Hospital Beijing China; ^3^ Chinese PLA Medical School Beijing China; ^4^ Innovation Research Center for Diabetic Foot West China Hospital, Sichuan University Chengdu China; ^5^ PLA Key Laboratory of Tissue Repair and Regenerative Medicine Beijing China; ^6^ Beijing Key Laboratory of Micro‐Nano Energy and Sensor, Center for High‐Entropy Energy and Systems Beijing Institute of Nanoenergy and Nanosystems, Chinese Academy of Sciences Beijing China

**Keywords:** diabetic wound healing, ellagic acid, fibroblasts, GelMA hydrogel, keratinocytes, small extracellular vesicles

## Abstract

Delayed diabetic wound healing is partially attributed to the functional disorder of skin repair cells caused by high glucose (HG). Small extracellular vehicles (sEVs) loaded with small‐molecule drugs represent a highly promising therapeutic strategy. This study aims to evaluate the therapeutic efficacy of ellagic acid‐encapsulated small extracellular vesicles (EA‐sEVs) in diabetic wound regeneration and to unravel related mechanisms. Cytotoxicity tests of ellagic acid (EA) as liposomal small molecules (LSMs) were performed with the CCK8 assay. EA was incorporated into sEVs obtained from chorionic plate‐mesenchymal stem cells (CP‐MSCs) to construct EA‐engineered sEVs. The protective effects of EA‐sEVs on human dermal fibroblasts (HDFs) and human epidermal keratinocytes (HEKs) induced by high glucose (HG) were assessed through the evaluation of their proliferative, migrative and differentiative capabilities. Furthermore, to illustrate the underlying mechanism, the specific biological targets of EA were predicted and confirmed. Finally, EA‐sEVs were encapsulated in GelMA hydrogel for investigating the pro‐healing effects on diabetic wounds. EA was harmless to cell viability, increasing the possibility and safety of drug development. EA‐engineered sEVs were fabricated by loading EA in sEVs. In vitro, EA‐sEVs promoted the proliferation, migration, and transdifferentiation of HG‐HDFs and the proliferation and migration of HG‐HEKs. Mechanism analysis elucidated that epidermal growth factor receptor (EGFR) was the specific biological target of EA. EA interacting with EGFR was responsible for the functional improvement of HG‐HDFs and HG‐HEKs. In vivo, EA‐sEVs encapsulated in GelMA promoted the healing of diabetic wounds by improving re‐epithelialisation, collagen formation and the expression of EGFR. Gel‐EA‐sEVs promoted diabetic wound healing by improving biological functions of HDFs and HEKs. EGFR was first identified as the specific biological target of EA and was responsible for the functional improvement of HG‐HDFs and HG‐HEKs by Gel‐EA‐sEVs. Hence, Gel‐EA‐sEVs can serve as a new promising active dressing for diabetic wound treatment.

## Graphical abstract

Ellagic acid (EA) was loaded in small extracellular vehicles (sEVs) derived from chorionic plate‐mesenchymal stem cells (CP‐MSCs) and then encapsulated in GelMA to construct Gel‐EA‐sEVs, which can be a promising treatment approach for diabetic wounds by improving biological functions of skin repair cells.

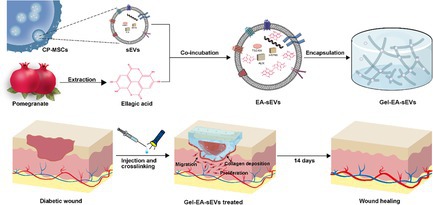

## 1 Introduction

With the increasing aging population, the incidence of diabetic chronic wounds is rising significantly, imposing a substantial economic strain on both families and society. For example, diabetic foot ulcers (DFU) are the typical representatives of diabetic wounds. DFU have resulted in 80% of lower extremity amputations among diabetics, increasing the risk of death [1]. The pathological mechanism of diabetic wounds is complex and poorly understood. Accumulating evidence suggests that hyperglycaemia injures the function of skin repair cells including fibroblasts and keratinocytes, resulting in the occurrence of diabetic chronic wounds [2, 3]. Therefore, developing effective therapeutic strategies to improve injured cellular function would be helpful for diabetic wound management.

Recently, the potential small‐molecule drugs have been discovered and developed for disease treatment by interacting with specific biological targets. Ellagic acid (EA) as a small molecule is the most active and abundant therapeutic compound in pomegranates. EA has been reported to exhibit a range of therapeutic benefits, including antiallergic, anticancer, hepatoprotective, nephroprotective, cardioprotective and neuroprotective effects [4], but the regenerative effects on diabetic wounds [5, 6, 7] and repair cells [8] are needed to be confirmed. Besides, EA is a liposomal small molecule with poor water solubility and low transdermal efficacy [9] which limited its practical application in disease treatments. To overcome these limitations, liposomes are synthesised for delivering small‐molecule drugs [10]. However, as artificial carriers, high concentrations of liposomes or prolonged exposure often increase cellular toxicity [11, 12]. Therefore, the more natural and safer vectors for EA delivery are needed to be developed for clinical translation.

Small extracellular vehicles are nano‐sized phospholipid bilayer‐enclosed vesicles secreted by all types of cells [13]. SEVs play a significant role in cell‐to‐cell communication and are crucial in many physiological and pathological processes [14, 15]. At present, mesenchymal stem cells (MSCs) are the primary source of sEVs. Recent evidence shows that MSC‐derived sEVs can effectively promote wound healing [16, 17, 18, 19, 20]. Chorionic plate‐mesenchymal stem cells (CP‐MSCs) are multipotent adult stem cells isolated from the human placental chorionic plate [21]. They possess capabilities such as self‐renewal, differentiation, homing and secretion of growth factors [22, 23], along with advantages including easy accessibility and minimal ethical constraints. These cells have demonstrated therapeutic efficacy in treating conditions such as acute pancreatitis [24], liver injury [25], optic nerve injury [26] and ovarian dysfunction [27]. However, there is a lack of research on sEVs originating from CP‐MSCs in diabetic wound treatments. As natural vehicles, native sEVs can deliver endogenous bioactive molecules such as microRNAs for wound treatments [28, 29]. However, the native sEVs commonly have limited therapeutic effects due to containing limited active components. Therefore, modifying sEVs to transfer exogenous payloads has been focused on for disease treatments [30, 31], suggesting that EA‐loaded sEVs may have promising therapeutic effects on diabetic wounds.

Additionally, sEVs as drug vectors still have some shortcomings including instability, low long‐term retention and a very short half‐life after transplantation in vivo. Loading sEVs in biomaterials [32] may be a powerful solution to sEV instability and realise a regulated and prolonged release of sEVs within a biological system. Gelatin methacryloyl (GelMA) hydrogel is a photosensitive hydrogel that is commonly utilised in tissue engineering applications due to its compatibility with biological tissues, biodegradability and low cost [33, 34]. Growing evidence demonstrated that the loose and porous structures of GelMA hydrogels are suitable for carrying sEVs, prolonging their retention for sustained release [35, 36]. Recently, sEVs loaded in GelMA hydrogels have been applied in wound treatments [19, 37].

In this study, sEVs were extracted from the supernatant of CP‐MSCs and then loaded with EA. High glucose‐induced human dermal fibroblasts (HG‐HDFs) and human epidermal keratinocytes (HG‐HEKs) were treated with EA‐sEVs and their abilities of proliferation, migration, transdifferentiation and collagen synthesis were evaluated. Next, to explore the underlying mechanism, the targets of EA were predicted and confirmed via four computational platforms and the blocking experiments. Subsequently, a new dressing for diabetic wounds was fabricated by loading EA‐sEVs in GelMA hydrogels. Finally, the therapeutic effects of Gel‐EA‐sEVs on diabetic wounds were observed and the related mechanisms were further explored.

## 2 Materials and Methods

### 2.1 Cell Culture

CP‐MSCs were donated by the team of Professor Sun Yuhong of the Western Theatre General Hospital. CP‐MSCs were cultured in MSC medium (Yocon, Beijing, China). The entire cell experiment process of CP‐MSCs was conducted in accordance with the latest set of guidelines on hMSC in China [38]. HDFs were acquired from the American Type Culture Collection (ATCC) (CRL‐4053, USA) and were cultured in Dulbecco's modified eagle medium (DMEM) (Gibco, USA), including 10% foetal bovine serum (FBS) (Gibco, USA) and 1% Penicillin–Streptomycin solution (Solarbio, Beijing, China). HEKs were obtained from ATCC and were cultured in EpiLife medium (Gibco, USA) enriched with 1% human keratinocyte growth supplement (HKGS, Gibco, USA). In the HG group, HDFs and HEKs were induced with 50 mM for 60 days and 25 mM glucose for 30 days, respectively.

### 2.2 Cell Counting Kit‐8 (CCK8) Proliferation Assay

The proliferation capability of cells and a cytotoxicity assessment of EA were performed on HG‐HDFs and HG‐HEKs by CCK8 assay (Beyotime, Shanghai, China). A total of 2 × 10^4^ HG‐HDFs or HG‐HEKs were plated in each well of 96‐well plates and subjected to various treatments for 24 h. Subsequently, CCK8 solutions were added into the culture medium. After an additional 2 h of incubation at 37°C, the absorbance at 450 nm was recorded using a microplate reader (SpectraMax M3, USA) for three repetitions.

### 2.3 Isolation of sEVs

SEVs were isolated from the supernatant of CP‐MSCs via ultracentrifugation following a previously described method [39]. Briefly, the collected supernatant underwent centrifugation at 500 g for a duration of 5 min, followed by filtration through a 0.22 μm filter to eliminate dead cells and cellular debris. A second centrifugation of the resulting supernatant was performed at 2000*g* for 30 min at a temperature of 4°C. Subsequent collection of the supernatant involved centrifugation at 10,000*g* for another 30 min at 4°C, during which the supernatant was discarded. The obtained pellet was resuspended in PBS and underwent a further centrifugation at 100,000*g* for 75 min. After resuspending in 1 mL of PBS, the remaining volume was used for characterisation and subsequent experiments, while the surplus EVs were stored at −80°C for future use. The entire extraction process complies with the guidelines for the production of stem cell‐derived extracellular vesicles [40].

### 2.4 Preparation and Identification of EA‐sEVs

EA (> 99% purity) was purchased from Med Chem Express (MCE, Shanghai, China). The sEVs (2 × 10^10^ particles/mL) were incubated with different concentrations of EA (50, 100, 200, 300, and 400 μM) at a temperature of 37°C for 1 h. The mixtures were centrifuged at 100,000*g* for 75 min to eliminate any unloaded EA. The precipitate was then re‐suspended with PBS and filtered through a 0.22 μm membrane for sterilisation.

To assess the loading efficiency of EA within sEVs, high‐performance liquid chromatography (HPLC, Agilent 1260, USA) was utilised, using a standard curve ranging from 300 to 9.375 μM, which exhibited strong linearity (*R* ^2^ = 0.9999). The analyses were performed at 30°C utilising a C18 column (250 mm ×4.6 mm, 5 μm, NanoChrom, Suzhou, China). A detection wavelength of 254 nm was set, with a solvent flow rate of 1 mL/min. The mobile phase was a mixture of acetonitrile (ACN) and H_2_O (containing 0.05% trifluoroacetic acid) with the solvent program outlined in Table S1. The particle size, concentration and morphology of natural sEVs and EA‐sEVs were detected with Flow NanoAnalyzer (Nanofcm, Xiamen, China) and transmission electron microscope (TEM, JEOL‐1400Flash, Japan). Western blot was employed to assess the presence of markers including CD63, CD9, tumour susceptibility gene101 (TSG101) and Calnexin.

### 2.5 Uptakes of sEVs

The isolated sEVs were incubated for 15 min at room temperature with Dil (Yeasen Biotechnology, Shanghai, China). Afterward, the labelled sEVs underwent ultracentrifugation at 100,000*g* for 75 min, followed by resuspension in PBS. Subsequently, HG‐HDFs (or HG‐HEKs) were combined with the labelled sEVs (2 × 10^10^ particles/mL) in a 48‐well plate for 6 h. The cells were then fixed using 4% paraformaldehyde (Solarbio, Beijing, China). The cytoskeleton was stained with phalloidin (Yeasen Biotechnology, Shanghai, China) while the nuclei were labelled with 4′,6‐diamidino‐2‐phenylindole (DAPI). Observations were made using a confocal microscope (Leica, Germany).

### 2.6 Cell Migration Assays

HG‐HDFs (or HG‐HEKs) were co‐cultured with sEVs (2 × 10^10^ particles/mL), EA (50 μM) or EA‐sEVs (2 × 10^10^ particles/mL) for 48 h. As for the blocking experiments of erlotinib, HG‐HDFs were co‐cultured with sEVs (2 × 10^10^ particles/mL) or EA‐sEVs (2 × 10^10^ particles/mL) with or without the co‐treatment of erlotinib (2 μM) for 48 h.

#### 2.6.1 Scratch Assay

HG‐HDFs or HG‐HEKs (4 × 10^5^ cells per well) with different treatments were seeded in a 6‐well plate and cultured to 90% confluence. A pipette tip (1 mL) was used to create a scratch. Images were captured at 0, 12 and 24 h with a microscope. The migration area was measured by Image J software.

#### 2.6.2 Transwell Assay

In the upper chambers of 24‐well plates (8 μm pore size, Corning Incorporated, USA), 0.2 mL of serum‐free medium containing HG‐HDFs or HG‐HEKs (4 × 10^4^ cells per well) was introduced, while the complete medium was supplied to the lower chambers. After a 24‐h period, the non‐migrated cells were removed from the upper membrane, and the cells that migrated to the lower surface were stained with crystal violet (Beyotime, Shanghai, China) and subsequently quantified using Image J software.

### 2.7 EdU Assay

Cells' proliferation ability was determined utilising BeyoClick EdU Cell Proliferation Kit with Alexa Fluor 594 (Beyotime, Shanghai, China), in accordance with the guidelines provided by the manufacturer. Briefly, HG‐HDFs and HG‐HEKs were plated into 24‐well plates for 24 h and then co‐incubated with EdU for 3 h (HG‐HDFs) or 12 h (HG‐HEKs). Following this incubation, the cells were fixed with 4% paraformaldehyde for 30 min and then permeabilised with 0.3% Triton X‐100 (Solarbio, Beijing, China) for 10 min. Subsequently, a click reaction mixture was applied for 30 min, and the samples were then stained with Hoechst 33342 for an additional 10 min. Representative images were captured through a fluorescence microscope (Nikon Instruments Inc., Japan).

### 2.8 Cell Immunofluorescence Staining

Firstly, HG‐HDFs were co‐cultured with sEVs (2 × 10^10^ particles/mL), EA (50 μM) or EA‐sEVs (2 × 10^10^ particles/mL) for 48 h. As for the blocking experiments of erlotinib, HG‐HDFs were co‐cultured with sEVs (2 × 10^10^ particles/mL) or EA‐sEVs (2 × 10^10^ particles/mL) with or without the co‐treatment of erlotinib (2 μM) for 48 h. Then, HG‐HDFs with different treatments were fixed with 4% paraformaldehyde and permeabilised with 0.5% Triton X‐100 (Beyotime, Shanghai, China). After blocking with 1% bovine serum albumin (BSA, Solarbio, Beijing, China), the cells were incubated overnight at 4°C with primary anti‐α‐smooth muscle actin (α‐SMA) (1:200, Proteintech, Wuhan, China). The next step involved incubation with Alexa‐Fluor‐conjugated secondary antibodies (1:1000, Invitrogen, USA) at room temperature for 1 h. Finally, nuclei were labelled using DAPI staining, and a fluorescence microscope was utilised to capture representative images.

### 2.9 Western Blot Analysis

The proteins in experiments, including identifying sEVs and EA‐sEVs, exploring the regulating function and the underlying signal pathways of EA‐sEVs on HG‐HDFs and HG‐HEKs, were extracted by RIPA lysis buffer. Following extraction, the proteins were separated via polyacrylamide gel electrophoresis and subsequently electro‐transferred to a polyvinylidene fluoride membrane. The membrane underwent blocking with QuickBlock Blocking Buffer (Solarbio, Beijing, China) for a duration of 15 min, after which it was incubated overnight at 4°C with primary antibodies including anti‐CD63, anti‐CD9, anti‐TSG101, anti‐Calnexin, anti‐α‐SMA, anti‐COL1 (1:1000, Proteintech, Wuhan, China), anti‐EGFR, anti‐C‐SRC (1:5000, Proteintech, Wuhan, China) and anti‐INSR (1:2000, Proteintech, Wuhan, China). Anti‐α‐tubulin (1:20,000, Proteintech, Wuhan, China) served as the reference control. After three washing steps, the membrane was incubated for 1 h at room temperature with either horseradish peroxidase (HRP)‐conjugated Affinipure Goat Anti‐Rabbit IgG (1:5000, Proteintech, Wuhan, China) or HRP‐conjugated Affinipure Goat Anti‐Mouse IgG antibodies. BeyoECL Star kit (Beyotime, Shanghai, China) was used to detect the intensity of protein expression via the UVITEC Alliance MINI HD9 system (UVITEC, Britain). Results were analysed by ImageJ software and normalised against α‐tubulin.

### 2.10 qRT‐PCR Analysis

Firstly, HG‐HDFs were co‐cultured with sEVs (2 × 10^10^ particles/mL), EA (50 μM) or EA‐sEVs (2 × 10^10^ particles/mL) for 24 h. Then, RNAs inside HG‐HDFs were extracted by an RNA extraction kit from YI SHAN Biotechnology (Shanghai, China). The Evo M‐MLV RT‐PCR kit (Accurate Biotechnology, Changsha, China) was used for reverse transcription. For quantitative PCR, a kit from Accurate Biotechnology (Changsha, China) was employed with the ABIPRISMVR 7300 Sequence Detection System (Applied Biosystems, USA). The primer sequences can be found in Table S2.

### 2.11 Preparation and Characterisation of GelMA Hydrogels

GelMA was obtained from Engineering for Life Co. Ltd. (Suzhou, China). Different monomer concentrations of GelMA in PBS (5%, 10% and 15%) were prepared and subjected to sterilisation through a 0.22 μm filtration device. The GelMA solution was photo‐crosslinked by ultraviolet radiation for 10 s for gelation. The morphological structure of the freeze‐dried hydrogels was observed by scanning electron microscope (SEM, ZEISS Gemini300, Germany).

For the swelling property, GelMA hydrogels (10 mm in diameter and 5 mm in thickness) were placed in a sealed tube filled with PBS (pH = 7.4) at 37°C to mimic the wound exudate conditions. Subsequently, GelMA hydrogels were taken out from the tube at specific time points, and their mass was recorded after wiping off the remained PBS with filter paper. The swelling ratios of GelMA hydrogels were determined using the following formula: Swelling ratio (%) = (*W*_*t*_ − *W*_0_)/*W*_0_ × 100%. Where W_t_ denotes the weight of GelMA hydrogels after swelling for a specific time and W_0_ denotes the initial weight of GelMA hydrogels.

For the degradation property, pieces of the swollen GelMA hydrogels were incubated in PBS containing type II collagenase (0.02 U/mL) at 37°C to mimic the wound conditions. The hydrogels were removed at specific time points and their mass was recorded after wiping off the PBS with filter paper until the hydrogels were completely biodegraded. The degradation ratio was determined using the formula below: Degradation ratio (%) = *W*_*d*_/*W*_*s*_ × 100%; where *W*_*d*_ denotes the weight of the hydrogels after degradation for a specific time and *W*_0_ denotes the initial weight of the hydrogels.

For the tensile tests, cuboid specimens of GelMA hydrogels, measuring 50 mm in length, 10 mm in width and 3 mm in thickness, were created. These samples underwent tensile testing using a universal testing machine (CMT6103, China) at a speed of 2 mm/min.

For biocompatibility evaluation, a cytotoxicity test was performed by CCK8 assay as above. Additionally, the haemolysis test was also performed to observe haemocompatibility properties. Briefly, the whole blood samples were collected in centrifuge tubes containing ddH_2_O, PBS, 5% Gel, 10% Gel or 15% Gel. After incubation at 37°C for 2 h, the blood samples were photographed and then centrifuged at 1000 rpm for 10 min. The supernatant was transferred into a 96‐well plate with 100 μL per well, and the absorbance was measured at 540 nm with a microplate reader.

### 2.12 Preparation and Characterisation of Gel‐EA‐sEVs

EA‐sEVs (4 × 10^9^ particles) were added into GelMA hydrogels (200 μL) with the above three concentrations to obtain the 5% Gel‐EA‐sEVs, 10% Gel‐EA‐sEVs and 15% Gel‐EA‐sEVs. For the release evaluation of EA‐sEVs, Gel‐EA‐sEVs were placed in a 24‐well plate with 1 mL of PBS. The supernatant was collected daily for a duration of 15 days. A BCA protein assay kit (Solarbio, Beijing, China) was utilised to determine the concentration of EA‐sEVs in the supernatant.

To observe the uptake of EA‐sEVs released from Gel‐EA‐sEVs, EA‐sEVs were labelled with Dil before adding them into 15% GelMA hydrogels, and a transwell assay filter (8 μm pore size, Corning Incorporated, USA) was used to create a co‐culture system with HG‐HDFs or HG‐HEKs (4 × 10^4^ cells per well) in the lower chamber and Gel‐EA‐sEVs in the upper chamber. After 12, 24, 48 and 72 h of coculture, the cytoskeleton was visualised using phalloidin (Yeasen Biotechnology, Shanghai, China), while DAPI was utilised for nuclear staining. A fluorescence microscope was employed to observe the immunofluorescence signals.

For biocompatibility evaluation of Gel‐EA‐sEVs, a live/dead staining assay was conducted using a cytotoxicity detection kit (Beyotime, Shanghai, China). In this procedure, HG‐HDFs or HG‐HEKs were plated into 24‐well plates and co‐incubated with or without 15% Gel‐EA‐sEVs for 72 h. Then, the cells were washed and subjected to staining with a mixture of Calcein‐AM (green fluorescent dye) and propidium iodide (red fluorescent dye). Representative images were captured using a fluorescence microscope (Nikon Instruments Inc., Japan). For observation of pathological injury, 15% Gel‐EA‐sEVs were applied on diabetic wounds; the major organs of mice including heart, liver, spleen, lung and kidney were harvested and stained with H&E as described previously.

### 2.13 Animal Experiments

Male DB/db mice aged 8 weeks were acquired from SPF Biotechnology Co. Ltd. (Beijing, China). The Animal Research Committee at PLA General Hospital in Beijing, China, approved the animal studies, and the mice were maintained under standard conditions for experimental animals. The mice were anaesthetised using sodium pentobarbital at a dose of 50 mg/kg. Full‐thickness skin wounds, each with a diameter of 10 mm, were created on the back of each mouse. The mice were then randomly assigned to one of six treatment groups (*n* = 6): PBS, EA, sEVs, EA‐sEVs, GelMA and Gel‐EA‐sEVs. PBS, EA (50 μM), sEVs (2 × 10^9^ particles/mL) and EA‐sEVs (50 μM, 2 × 10^9^ particle/mL) were administered subcutaneously by a micro syringe (Hamilton, Switzerland) at four distinct sites around the wounds, with 25 μL per site, repeated every 3 days. 100 μL GelMA and 100 μL Gel‐EA‐sEVs (50 μM, 2 × 10^9^ particle/mL) were dropped on the wounds and photo‐crosslinked by ultraviolet radiation for 20 s after surgery. Wounds were recorded and assessed using image J software on days 0, 3, 7, 14 and 21. Subsequently, the mice were sacrificed, and the wound tissues were harvested at the indicated times for further use.

### 2.14 Histological Examination

The wound tissues with different treatments were fixed with 4% paraformaldehyde for at least 24 h, then dehydrated gradually and embedded in paraffin. The tissues were cut into 4 μm thick slices and stained with haematoxylin and eosin (H&E) kit (Solarbio, Beijing, China) and Masson's kit (Solarbio, Beijing, China). The images were observed and obtained using a Confocal Laser Scanning Microscope (CLSM, Olympus, Japan). The wound length and wound area were measured as described previously [41].

### 2.15 Immunohistochemistry and Immunofluorescence Staining of Wound Tissues

For immunohistochemical analysis, tissue sections were deparaffinised and hydrated. The activity of endogenous peroxidase was inhibited by treating the sections with 3% hydrogen peroxide for 10 min, followed by antigen retrieval. Slices were blocked with 2% BSA for 1 h and incubated overnight at 4°C with primary anti‐α‐SMA, anti‐COL1A, anti‐EGFR (1:200, Proteintech, Wuhan, China). Slices were incubated with HRP‐conjugated secondary antibody for 1 h at room temperature and subsequently stained with DAB substrate solution (Proteintech, Wuhan, China). Additionally, immunofluorescence staining was also performed by incubating with the above primary antibodies, followed by Alexa‐Fluor‐conjugated secondary antibodies (1:1000, Invitrogen, USA) and DAPI. The images were captured using a CLSM.

### 2.16 Statistical Analysis

All data were analysed with SPSS software and are presented as mean ± standard deviation (SD). Comparisons between two groups were conducted using Student's *t*‐test, while one‐way analysis of variance (ANOVA) was employed for multigroup comparisons. The *p* value < 0.05 was deemed statistically significant.

## 3 Results

### 3.1 Preparation and Characterisation of sEVs and EA‐sEVs

**FIGURE 1:**
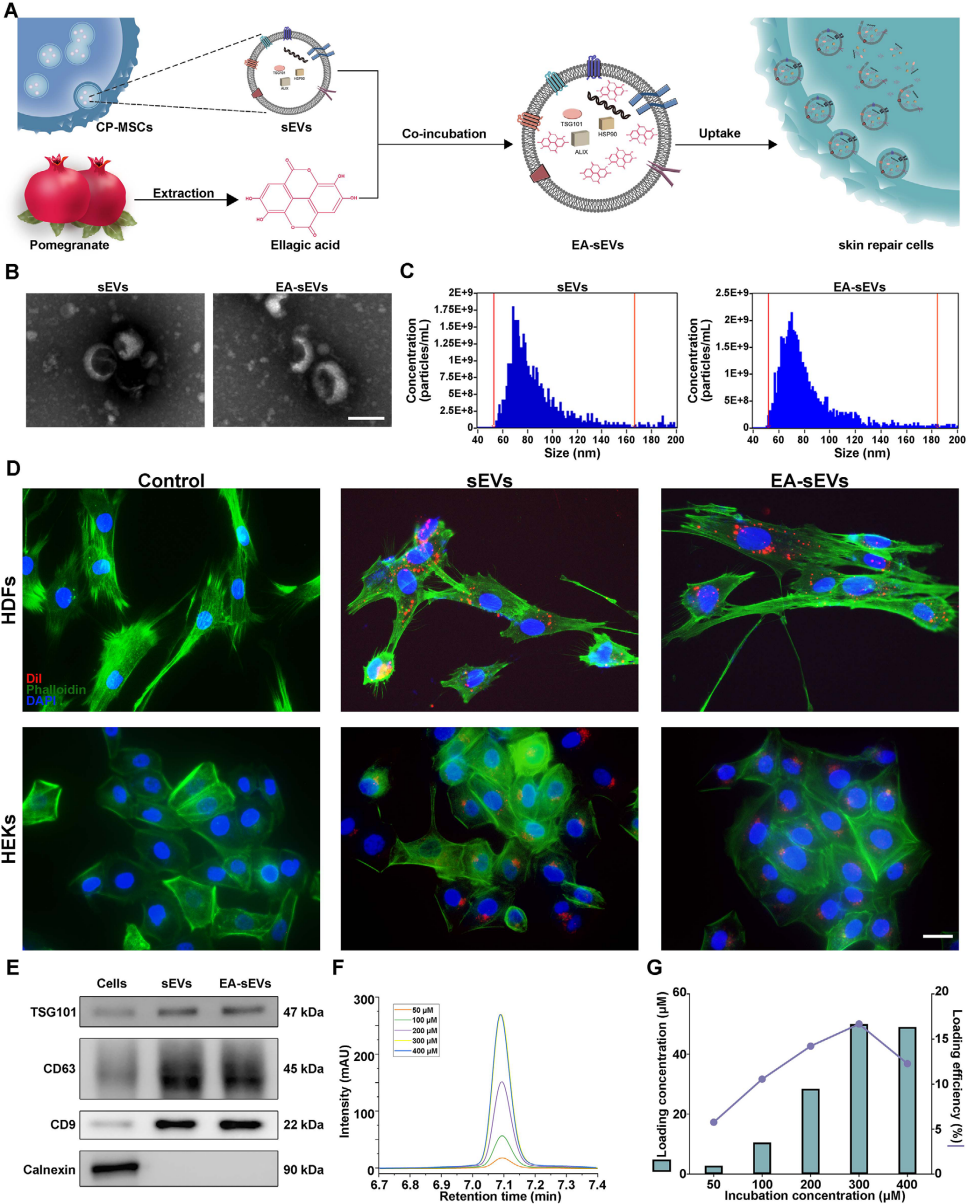
Preparation and characterisation of EA‐sEVs. (A) Schematic overview of preparation procedure of the EA‐sEVs and their internalisation by skin repair cells. (B) TEM images of the isolated sEVs and EA‐sEVs; scale bar, 100 nm. (C) Size distribution and concentration of the isolated sEVs and EA‐sEVs measured by flow cytometry. (D) Representative fluorescent images showing the internalisation of Dil‐labelled EVs and EA‐sEVs by HG‐HDFs and HG‐HEKs at 6 h; scale bar, 50 μm. Blue: DAPI, red: Dil, green: Phalloidine. (E) Western blot analysis showing protein expressions of TSG101, CD63 and CD9 in CP‐MSCs, sEVs and EA‐sEVs. (F) HPLC analysis reflecting the amounts of EA loaded in sEVs after co‐incubation. (G) Loading concentration and efficiency of EA inside sEVs.

Before construction of EA‐sEVs, we performed the cytotoxicity test of EA on HG‐HDFs by CCK8 assay and found that EA barely affected cell viability (Figure S1). Subsequently, EA was incubated with sEVs from CP‐MSCs to acquire EA‐sEVs, and any unbound EA was eliminated through ultracentrifugation (Figure 1A). The morphological characteristics and size distribution of EA‐sEVs were analysed by TEM and Flow NanoAnalyzer, respectively. EA‐sEVs exhibited typical double‐layer cup‐shaped membranous structures and a comparable size distribution in diameter (average 80.25 vs. 83.35 nm) (Figure 1B,C) as that of natural sEVs. The surface markers such as CD63, CD9 and TSG101 were found to be highly expressed in both natural sEVs and EA‐sEVs, while Calnexin was only present in the cell lysis group (Figure 1E). Additionally, we also found that both natural sEVs and EA‐sEVs were successfully internalised by HD‐HDFs and HG‐HEKs (Figure 1D). To determine the loading efficiency, we calculated the concentration ratio of the loaded EA to the EA that was initially incubated. Results from HPLC demonstrated that the loading concentration of EA within sEVs incrementally increased with higher incubation concentrations until reaching the saturation point of a predetermined number of sEVs (2 × 10^10^ particles/mL). As shown in Figure 1F,G, the maximum loading concentration of EA inside sEVs was 50 μM and the highest loading efficiency was 16.67%.

### 3.2 EA‐sEVs Promoted the Proliferation, Migration and Myofibroblast Transdifferentiation of HG‐HDFs

**FIGURE 2:**
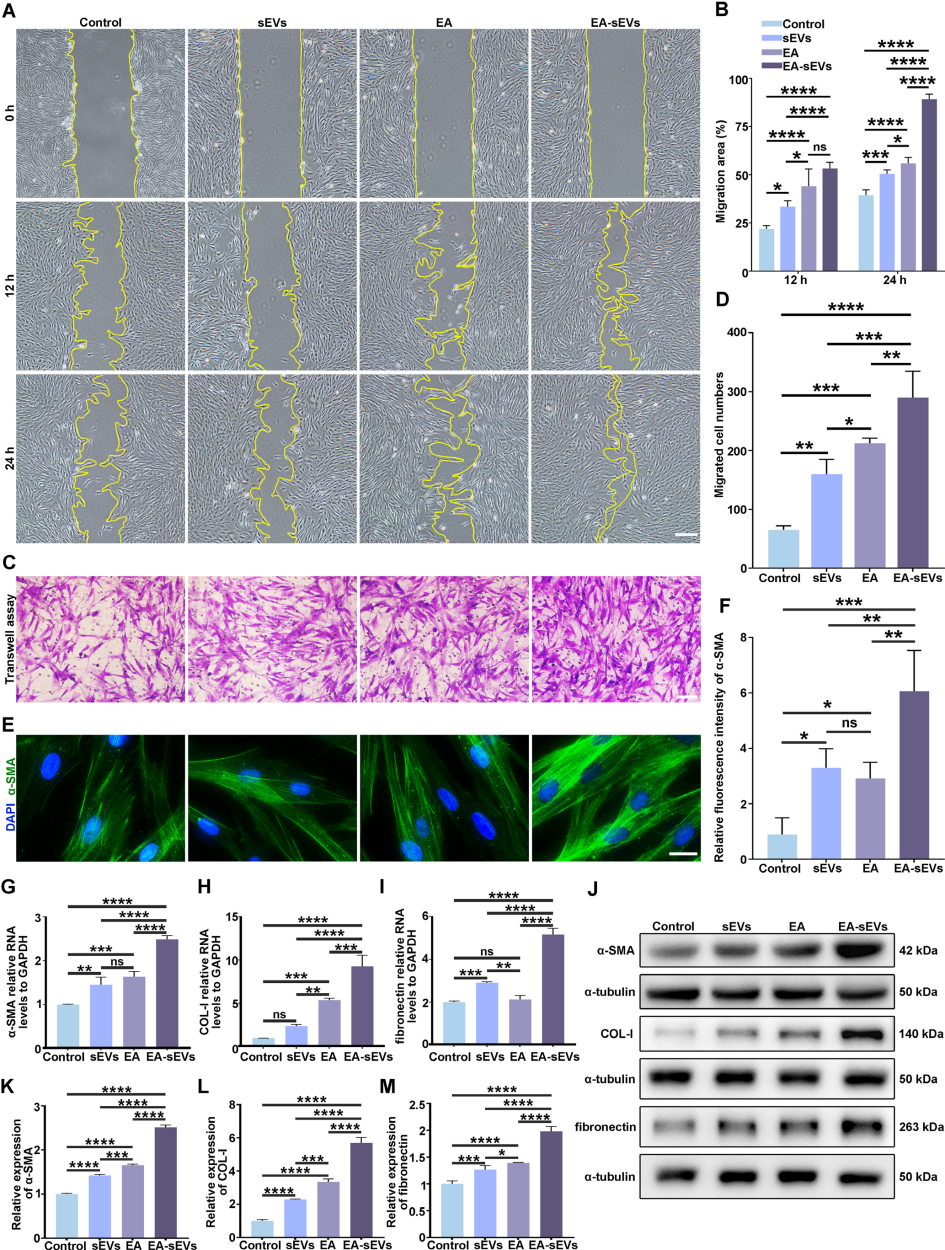
EA‐EVs promoted the migration and myofibroblast transdifferentiation of HG‐HDFs. (A) Migration ability of HG‐HDFs treated with sEVs, EA and EA‐sEVs reflected by scratch assay; scale bar, 50 μm. (B) Statistical analysis of (A). (C) Migration ability of HG‐HDFs treated with different groups demonstrated by transwell assay; scale bar, 50 μm. (D) Statistical analysis of (C). (E) Immunofluorescent staining images of α‐SMA inside HG‐HDFs treated with different groups; scale bar, 50 μm. (F) Quantitative analysis of (E). (G–I) qRT‐PCR results showing the mRNA levels of α‐SMA, COL‐1 and fibronectin in HG‐HDFs after treatments with sEVs, EA and EA‐sEVs. (J) Western blot analysis showing protein expressions of α‐SMA, COL‐1 and FN in HG‐HDFs with different treatments. (K‐M) Quantitative analysis of band intensities in (J). The data are presented as the mean ± SD. Differences among the groups were examined with one‐way ANOVA with Tukey's post‐test. *p < 0.05, **p < 0.01, ***p < 0.001, ****p < 0.0001, ns, not significant. *n* = 3 per group.

To observe the effects of EA‐sEVs on the functions of HG‐HDFs, a high glucose model of HDFs was created by culturing HDFs with 50 mM glucose for 60 days. HG‐HDFs were divided into four groups, including PBS (control), EA, sEVs and EA‐sEVs. EA and EA‐sEVs groups adopted the same EA concentration of 50 μM. sEVs and EA‐sEVs groups adopted the same sEV concentration of 2 × 10^10^ particles/mL. The proliferative capability of HG‐HDFs under various treatments was assessed using EdU and CCK8 assays. As shown in Figure S3A,B, a greater proportion of EdU‐positive cells was exhibited in the EA, sEVs and EA‐sEVs groups compared with that in the control group, with the greatest proportion shown in the EA‐sEVs group. The CCK8 assay demonstrated the consistent results, indicating that EA‐sEV treatment significantly enhanced the proliferation of HG‐HEKs (Figure S3C). The migration capability of HG‐HDFs was evaluated by scratch and transwell assays. Findings from the scratch assay indicated that the migration area of HG‐HDFs in the EA‐sEVs‐treated group was significantly greater than that in the other groups at both 12 and 24 h. The migration area in EA and sEVs groups was also higher compared with the control group (Figure 2A,B). Additionally, the same results were observed by transwell assays (Figure 2C,D). Subsequently, we investigated the promotive effects of EA‐sEVs on the transdifferentiation of HG‐HDFs by detecting the expression of myofibroblast marker α‐SMA and secretory proteins type I collagen (COL‐1) and fibronectin (FN) in EA‐sEV‐treated HG‐HDFs. Compared with the other three groups, EA‐sEVs upregulated α‐SMA protein expression significantly (Figure 2E,F). Furthermore, aligning with the aforementioned results, the expressions of COL‐1 and FN were markedly enhanced in the EA‐sEV‐treated group (Figure 2G–M). Collectively, these results indicated that EA‐sEVs promoted the abilities of migration and myofibroblast transdifferentiation of HG‐HDFs.

### 3.3 EA‐sEVs Promoted the Proliferation and Migration of HG‐HEKs

**FIGURE 3:**
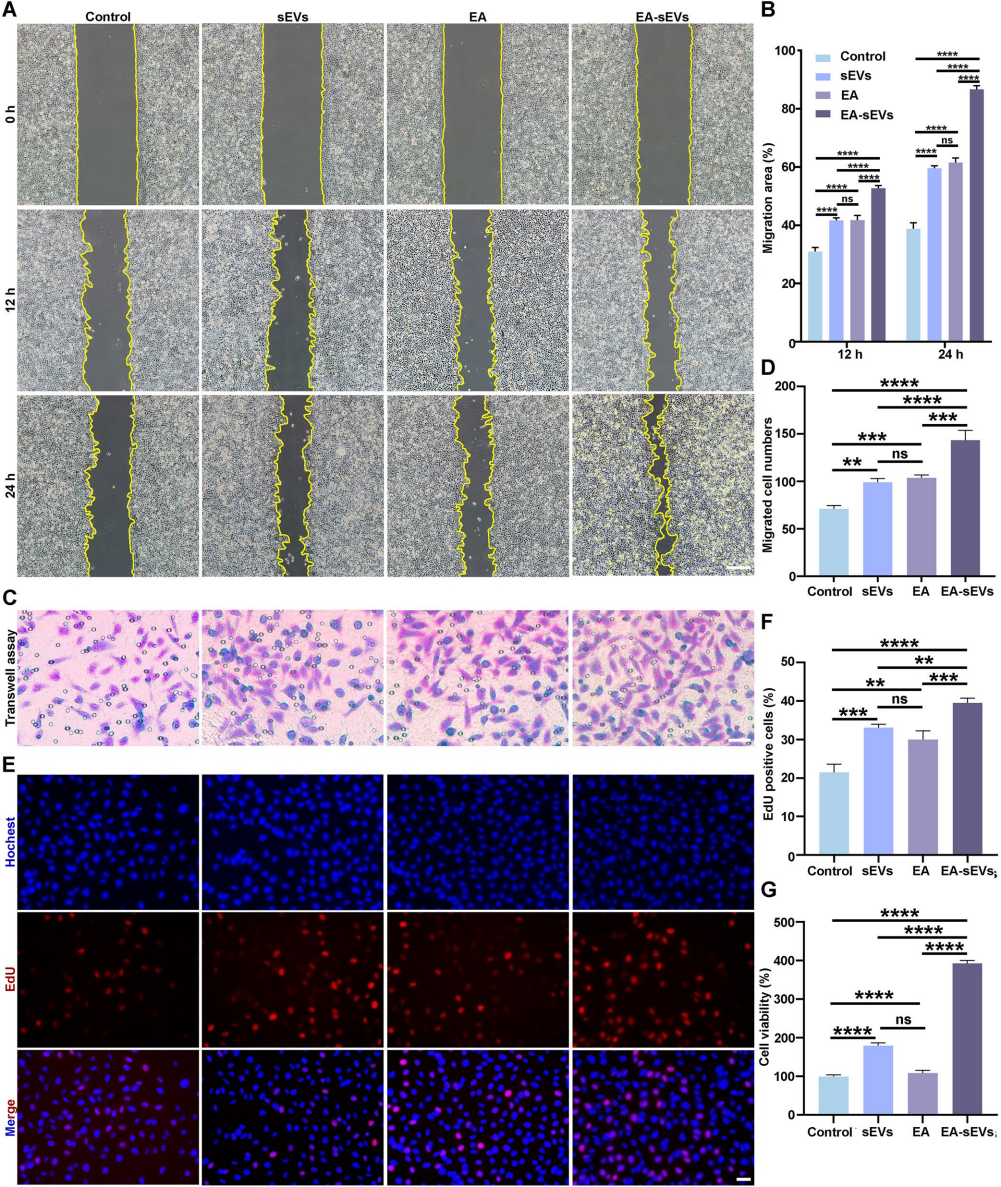
EA‐EVs promoted the proliferation and migration of HG‐HEKs. (A) Migration ability of HG‐HEKs treated with sEVs, EA and EA‐sEVs reflected by scratch assay; scale bar, 50 μm. (B) Statistical analysis of (A) (*n* = 3). (C) Migration ability of HG‐HEKs treated with different groups demonstrated by transwell assay; scale bar, 50 μm. (D) Statistical analysis of (C) (*n* = 3). (E) EdU immunofluorescent staining images of HG‐HEKs treated with different groups; scale bar, 50 μm. (F) Quantitative analysis of (E) (*n* = 3). (G) The CCK8 results of HG‐HEKs treated with different groups (*n* = 6). The data are presented as the mean ± SD. Differences among the groups were examined with one‐way ANOVA with Tukey's post‐test. *p < 0.05, **p < 0.01, ***p < 0.001, ****p < 0.0001, ns, not significant.

To delve deeper into the therapeutic potential of EA‐sEVs for diabetic wound healing, we investigated the impact of EA‐sEVs on the proliferation and migration of HG‐HEKs. The proliferative capability of HG‐HEKs under various treatments was assessed using EdU and CCK8 assays. As shown in Figure 3E,F, a greater proportion of EdU‐positive cells was exhibited in the EA, sEVs and EA‐sEVs groups compared with that in the control group, with the greatest proportion shown in the EA‐sEVs group. The CCK8 assay demonstrated consistent results, indicating that EA‐sEV treatment significantly enhanced the proliferation of HG‐HEKs (Figure 3G). Scratch and transwell assays were utilised to evaluate the impact of EA‐sEVs on the migratory capability of HG‐HEKs. In the scratch experiment, the migration area of the EA‐sEVs group was significantly larger compared with the other groups, indicating a well pronounced enhancement in cell migration (Figure 3A,B). Supporting the findings from the scratch assay, the transwell assay revealed that EA‐sEVs enhanced the count of migrated cells (Figure 3C,D). These results suggested that EA‐sEVs had positive effects on the proliferation and migration of HG‐HEK.

### 3.4 EGFR Was Responsible for Improving the Cellular Function by EA‐sEVs

**FIGURE 4:**
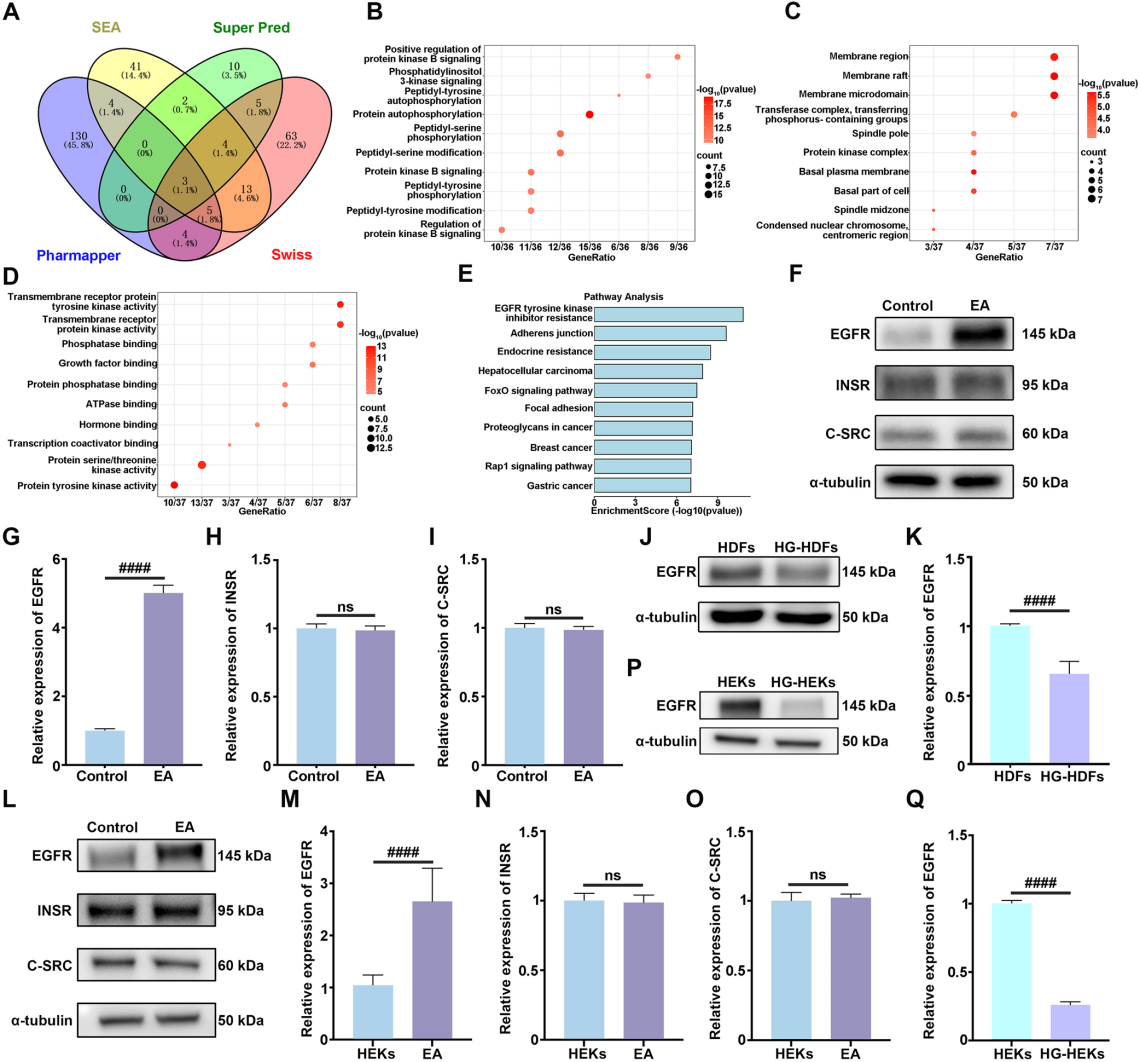
EGFR was identified as a target protein of EA. (A) Target proteins of EA were predicted by SwissTargetPrediction, Super Pred, SEA and Pharmmapper database and three proteins were screened out. (B‐D) GO enrichment analysis results were classified according to BP, CC and MF. The top 10 GO term items with the minimum *p* value and the most significant enrichment in each GO category were selected. (E) KEGG pathway enrichment analysis of EA. The top 10 KEGG pathways with the smallest *p* value were selected. (F) Western blot analysis showing the expression levels of EGFR, INSR and C‐SRC in HG‐HDFs stimulated with EA after 48 h. (G–I) Statistical analysis of (F). (J) Representative Western blot images of EGFR in HDFs and HG‐HDFs. (K) Statistical analysis of (J). (L) Western blot analysis showing the expression levels of EGFR, INSR and C‐SRC in HG‐HEKs stimulated with EA after 48 h. (M–O) Statistical analysis of (L). (P) Representative Western blot images of EGFR in HEKs and HG‐HEKs. (Q) Statistical analysis of (P). Data were analysed using one‐way ANOVA with Tukey's post‐test and presented as mean ± SD. ^#^ *p* < 0.05 ^*##*^ *p* < 0.01, ^*###*^ *p* < 0.001, ^*####*^ *p* < 0.0001, ns, not significant. *n* = 3 per group.

To illustrate the mechanism of EA‐sEVs‐induced function improvement of HG‐HDFs and HG‐HEKs, four computational platforms (SwissTargetPrediction, Super Pred, SEA, Pharmmapper) were used to predict the molecular targets of EA. As shown in the Venn diagram, the number of targets predicted by Super Pred is less than that predicted by the other three platforms (Figure 4A). Due to the different algorithms used by each platform, the prediction results were quite different. The common targets of EA on two platforms were collected to complete Gene Ontology (GO) analysis and Kyoto Encyclopedia of Genes and Genomes (KEGG) pathway enrichment analysis. The results of GO enrichment analysis were categorised based on biological process (BP), cell component (CC) and molecular function (MF). The 10 GO term entries exhibiting minimum *p* value and most significant enrichment in each GO category were selected. GO analysis demonstrated that the predicted intersecting targets of EA were mainly involved in biological processes such as positive regulation of protein kinase B signalling, phosphatidylinositol 3‐kinase signalling and peptidyl‐tyrosine autophosphorylation (Figure 4B), which were distributed in cellular locations such as membrane region, membrane raft and membrane microdomain (Figure 4C), and were involved in the molecular functions such as transmembrane receptor protein tyrosine kinase activity, transmembrane receptor protein kinase activity and phosphatase binding activity (Figure 4D). KEGG analysis revealed that the predicted intersecting targets of EA primarily participated in signalling pathways such as EGFR tyrosine kinase inhibitor resistance, adhesive junction and endocrine resistance (Figure 4E).

**FIGURE 5:**
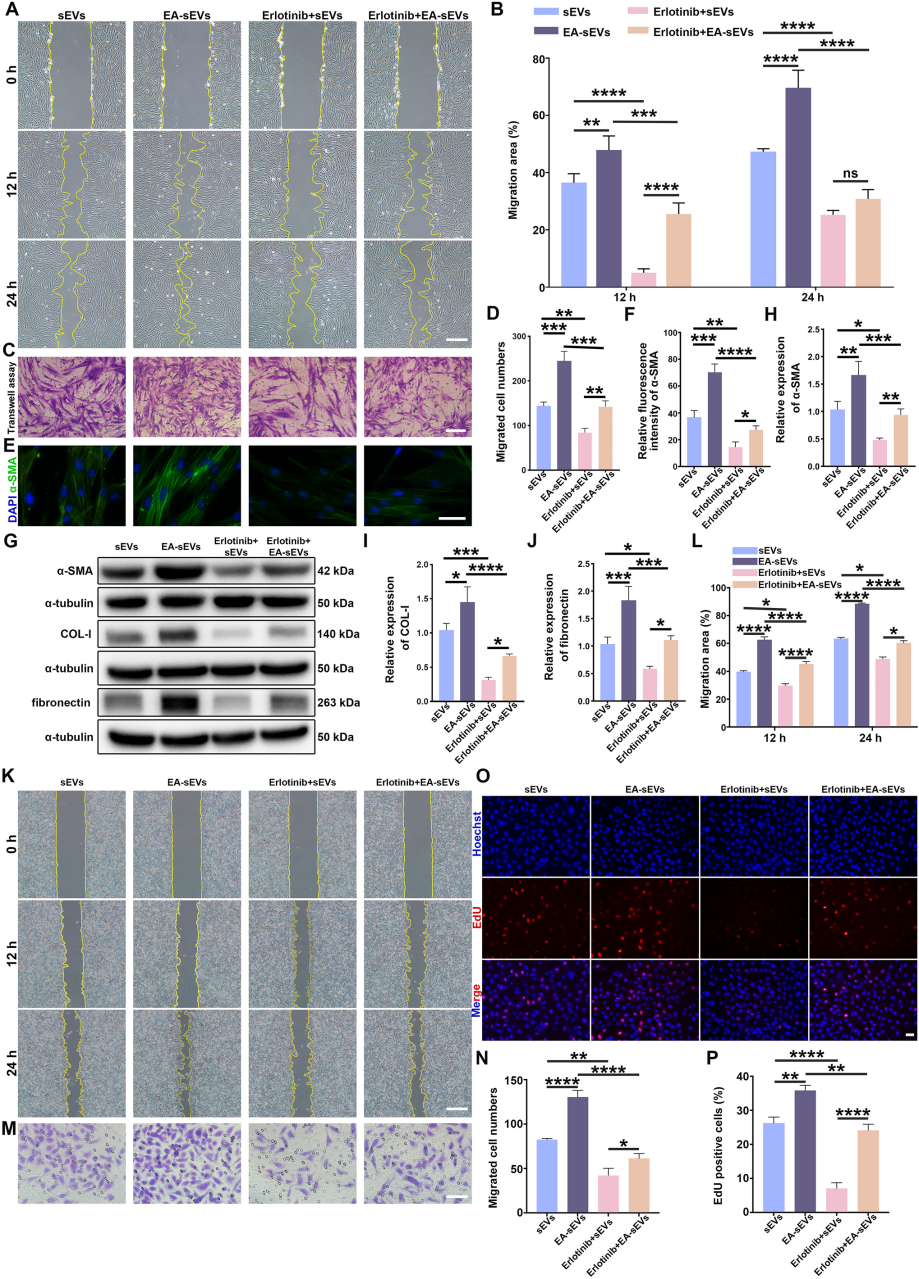
Erlotinib blocked the improved functions of HG‐HDFs and HG‐HEKs by EA‐sEVs. (A) Migration ability of HG‐HDFs with the treatments of sEVs, EA‐sEVs, erlotinib + sEVs and erlotinib + EA‐sEVs displayed by scratch assay; scale bar, 50 μm. (B) Quantitative results of (G). (C) Migration ability of HG‐HDFs following the indicated treatments analysed by transwell assay, scale bar, 50 μm. (D) Quantitative analysis of (I). (E) Immunofluorescent staining images of α‐SMA inside HG‐HDFs upon different treatments; scale bar, 50 μm. (F) Quantitative analysis of α‐SMA in (E). (G) Western blot analysis showing the expression levels of α‐SMA, COL‐1 and FN in HG‐HDFs with different treatments. (H–J) Quantitative results of band intensities in (G). (K) Migration ability of HG‐HEKs treated with different groups reflected by scratch assay; scale bar, 50 μm. (L) Statistical analysis of (K). (M) Migration ability of HG‐HEKs following the indicated treatments analysed by transwell assay, scale bar, 50 μm. (N) Statistical analysis of (M). (O) EdU immunofluorescent staining images of HG‐HEKs treated with different groups; scale bar, 50 μm. (P) Statistical analysis of (O). The data are presented as the mean ± SD. Differences among the groups were examined with one‐way ANOVA with Tukey's post‐test. *p < 0.05, **p < 0.01, ***p < 0.001, ****p < 0.0001, ns, not significant. *n* = 3 per group.

Based on the prediction results, the expressions of three intersection targets including EGFR, INSR and C‐SRC in HG‐HDFs were further confirmed by western blot analysis (Figure 4F–I). The results indicated that the expression of EGFR in HG‐HDFs was markedly increased after treatment with EA for 48 h and the expression of EGFR in HG‐HDFs was lower than that in HDFs (Figure 4J,K). However, the treatment of EA did not significantly affect the expression levels of INSR and C‐SRC in HG‐HDFs. Similar experimental results were observed in HG‐HEKs cells (Figure 4L–Q). Next, to further verify the role of EGFR in the functional regulation of HG‐HDFs and HG‐HEKs by EA‐sEVs, EGFR inhibitor (erlotinib) was used to block the activity of EGFR. The results of scratch and transwell assays indicated that erlotinib effectively blocked the enhanced migratory capacity of HG‐HDFs (Figure 5A–D) and HG‐HEKs (Figure 5K–N) induced by EA‐sEVs. Furthermore, the expressions of α‐SMA, COL‐1 and FN in EA‐sEVs‐treated HG‐HDFs were decreased by erlotinib (Figure 5E–J). The results of the EdU assay indicated that erlotinib inhibited the proliferative effects of EA on HG‐HDFs and HG‐HEKs (Figures S4 and 5O,P).

In summary, EGFR was confirmed as the target protein of EA. The expression and activation of EGFR were responsible for improving the functions of HG‐HDFs and HG‐HEKs by EA‐sEVs.

### 3.5 Preparation and Characterisation of GelMA Hydrogels and Gel‐EA‐sEVs

**FIGURE 6:**
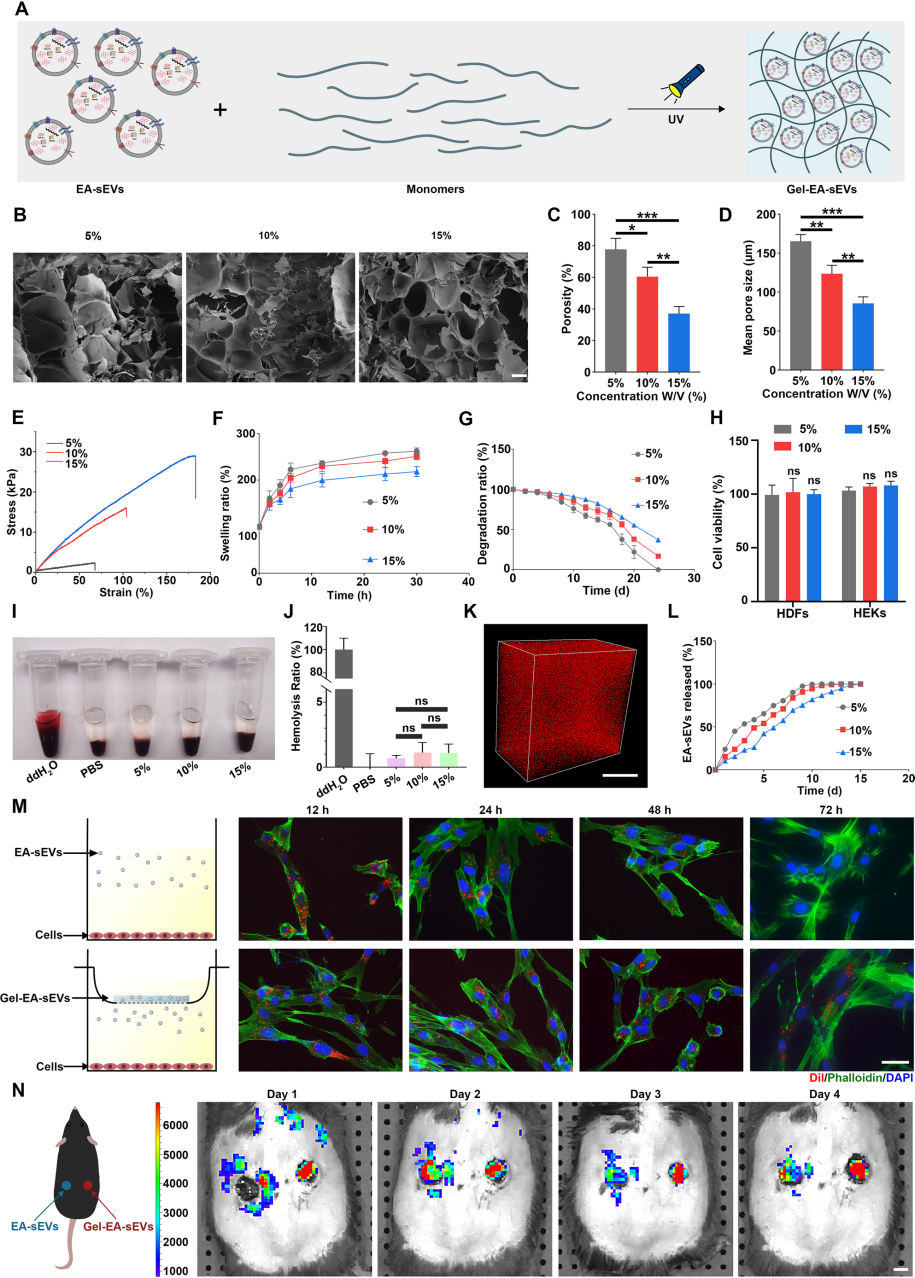
Characterisation of GelMA and Gel‐EA‐sEVs. (A) Schematic displaying the synthesis process of Gel‐EA‐sEVs. (B) SEM images of GelMA hydrogels with different monomer concentrations; scale bar, 100 μm. (C–G) Porosity (%), mean pore size, stress–strain curves, swelling behaviour and degradation ratio of GelMA hydrogels with different monomer concentrations. (H) Cell viability of HG‐HDFs and HG‐HEKs cultured on GelMA hydrogels with different concentrations. (I, J) Haemocompatibility and haemolysis ratio of GelMA hydrogels with different concentrations. (K) CLSM image showing the uniform distribution of EA‐sEVs in GelMA hydrogel; scale bar, 20 μm. (L) Released curve of EA‐sEVs from GelMA hydrogels with different monomer concentrations. (M) Illustration and fluorescent images reflecting the release time of EA‐sEVs in free form or encapsulation form inside HG‐HDFs; scale bar, 50 μm. Blue: DAPI, red: Dil, green: Phalloidine. (N) Illustration and representative images reflecting retention time of Dil labelled EA‐sEVs in free form or encapsulation form at wound area; scale bar, 5 mm. *n* = 3 per group.

GelMA hydrogels with different concentrations were prepared for encapsulating EA‐sEVs (Figure 6A). First, the porous structures of GelMA hydrogels at 5%, 10% and 15% concentrations were observed by SEM (Figure 6B). With the increasing concentrations, the porosity and pore size of GelMA hydrogels were gradually decreased (Figure 6C,D). Higher tensile strength was achieved in 15% GelMA hydrogels (Figure 6E). As shown in Figure 6F, all hydrogels reached swelling equilibrium around 30 h. However, the swelling ratio of 15% GelMA hydrogels was lower than that of other concentrations of GelMA hydrogels, likely due to the decreased porosity and pore size. Additionally, the degradation ratio of 15% GelMA hydrogels was much lower than that of low concentration groups (Figure 6G). To assess the biocompatibility of GelMA hydrogels, CCK8 and haemolysis assays were conducted. The CCK8 assay results indicated that the viability of HG‐HDFs and HG‐HEKs was not affected (Figure 6H). The supernatant from the hydrolysis of GelMA hydrogels with different concentrations exhibited a similar colour to that of the control (Figure 6I). Quantitative analysis revealed that the haemolysis rates for all concentrations were < 5% (Figure 6J), indicating that GelMA hydrogels of all concentrations possess good haemocompatibility [42].

Next, EA‐sEVs were encapsulated in GelMA hydrogels at different concentrations (5%, 10% and 15%). CLSM images indicated the successful loading and uniform distribution of EA‐sEVs in GelMA hydrogels (Figure 6K). The release behaviours of EA‐sEVs from GelMA hydrogels in vitro were also evaluated at varying concentrations, and the findings revealed that the 15% GelMA hydrogels exhibited a more stable and continuous release of EA‐sEVs compared with others (Figure 6L). Then we observed the continuous release time of EA‐sEVs from 15% GelMA hydrogels by culturing HG‐HDFs and HG‐HEKs with 15% Gel‐EA‐sEVs for 12, 24, 48 and 72 h. Figures 6M and S5 showed that the red fluorescence was still detectable after 72 h in the Gel‐EA‐sEV group, whereas that in the EA‐sEVs group became weak from 48 h and nearly disappeared at 72 h. The release time of Gel‐EA‐sEVs was also evaluated in vivo by live animal imaging. The results indicated that the fluorescence intensity of EA‐sEVs obviously was reduced on day 4 in the four‐point injection group, whereas that in the Gel‐EA‐sEVs group displayed no significant regression (Figure 6N). These findings suggested that GelMA hydrogels extended the release time of EA‐sEVs both in vitro and in vivo. The biocompatibility of Gel‐EA‐sEVs was also evaluated by H&E staining of key organs. The results showed no obvious histological abnormalities or inflammatory cell infiltration in the Gel‐EA‐sEVs group compared with the control group (Figure S2A). Live/dead staining assay revealed only a few dead cells in each group, with no notable difference in the number of viable cells between the control and Gel‐EA‐sEVs group (Figure S2B).

### 3.6 Gel‐EA‐sEVs Facilitated Diabetic Wound Healing With Enhanced Re‐Epithelialisation and Collagen Deposition by Activating EGFR

**FIGURE 7:**
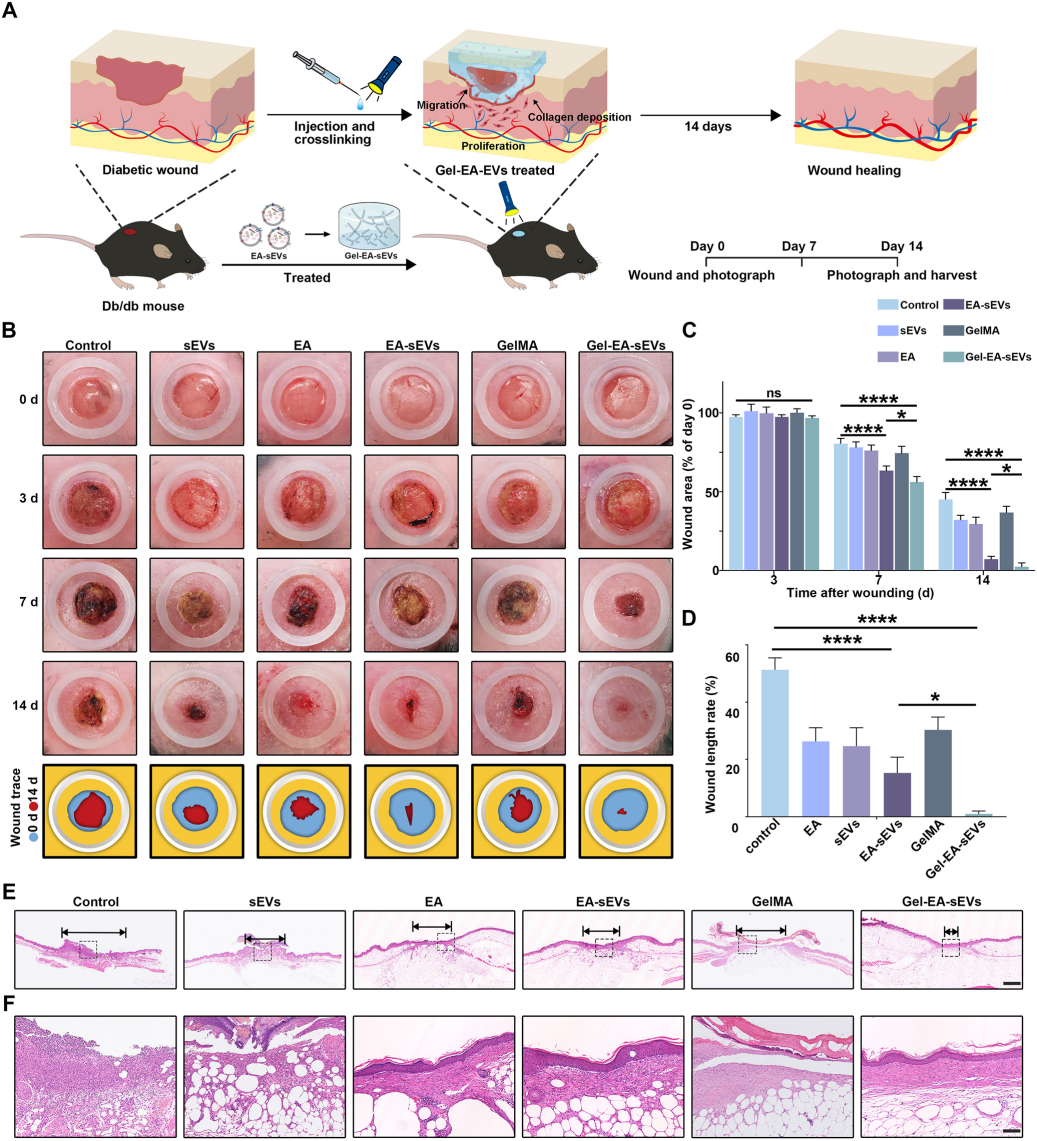
Gel‐EA‐sEVs enhanced diabetic wound healing. (A) Illustration showing the timeline of animal experiments and the pro‐healing effects of Gel‐EA‐sEVs on diabetic wounds. (B) Digital photographs of the wound areas on day 0, 3, 7 and 14 with the administration of PBS, sEVs, EA, EA‐sEVs, GelMA and Gel‐EA‐sEVs. (C) Quantitative analysis of the wound area in (B). (D) Quantitative analysis of the wound length rate. (E, F) Panoramic and enlarged views of H&E staining of wound sections on day 14 with different administration; scale bar, 1 mm for (E) and 100 μm for (F). The data are presented as the mean ± SD. Differences among the groups were examined with one‐way ANOVA with Tukey's post‐test. *p < 0.05, ***p < 0.001, ****p < 0.0001, ns, not significant. *n* = 6 per group.

To observe the therapeutic effects of Gel‐EA‐sEVs in vivo, the full‐thickness skin wounds were established on the backs of diabetic mice (1‐cm diameter), which were randomly assigned into six groups including control, sEVs, EA, EA‐sEVs, GelMA and Gel‐EA‐sEVs. The representative macroscopic images of the wounds in every group were taken on days 0, 3, 7 and 14 (Figure 7A). We observed that the treatment with sEVs, EA, EA‐sEVs, GelMA and Gel‐EA‐sEVs significantly reduced the wound area compared with control, and the wounds in the Gel‐EA‐sEV group were almost completely healed on day 14 (Figure 7B). By calculating the wound area, the Gel‐EA‐sEV group exhibited the smallest wound area at all time points, and the wound virtually closed on day 14 (Figure 7C).

**FIGURE 8:**
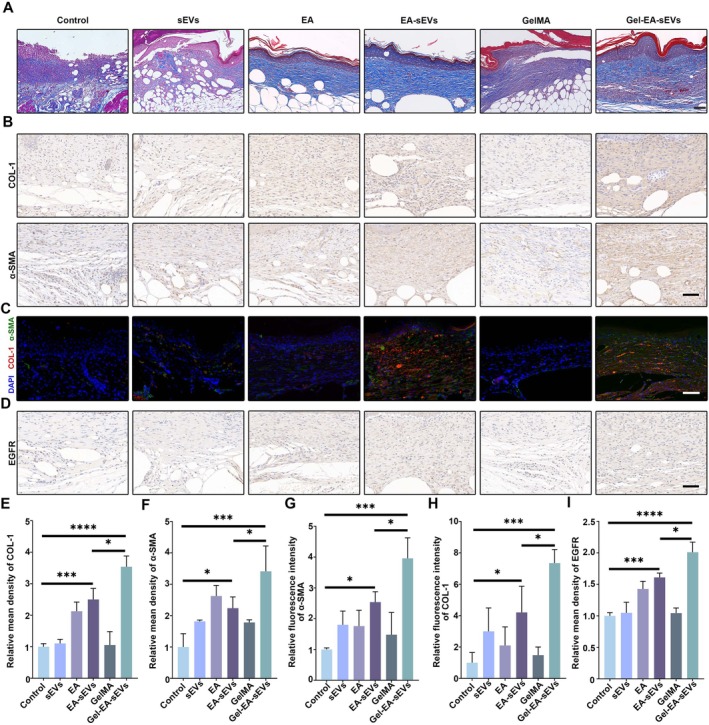
Gel‐EA‐sEVs promoted myofibroblast transdifferentiation and EGFR expression in vivo. (A) Representative images of Masson staining of wound sections on day 14 with different administration; scale bar, 100 μm. (B) Immunohistochemical staining images of α‐SMA and COL‐1 in diabetic wounds on day 14 with the treatments of PBS, sEVs, EA, EA‐sEVs, GelMA and Gel‐EA‐sEVs; scale bar, 200 μm. (C) Immunofluorescence staining images of α‐SMA and COL‐1 in diabetic wounds on day 14 with different treatments; scale bar, 200 μm. (D) Immunohistochemical staining images of EGFR in diabetic wounds on day 14 with different treatments; scale bar, 200 μm. (E, F) Quantification results of COL‐1 and α‐SMA in (B). (G, H) Quantification analysis of α‐SMA and COL‐1 in (C). (I) Quantification analysis of EGFR in (D). The data are presented as the mean ± SD. Differences among the groups were examined with one‐way ANOVA with Tukey's post‐test. *p < 0.05, ***p < 0.001, ****p < 0.0001. *n* = 6 per group.

Subsequently, the wound tissues were harvested from every group at day 14 for histology and molecular analysis. H&E staining was conducted to assess the re‐epithelialisation process in all wounds. We found well‐arranged epithelium and regularly distributed keratinocytes in the Gel‐EA‐sEVs group (Figure 7E,F). According to our quantitative analysis, the Gel‐EA‐sEVs group exhibited the lowest wound width rate (Figure 7D). Masson staining was performed to observe the collagen distribution in different groups. Collagen fibres in the Gel‐EA‐sEVs group displayed a denser, thicker and better deposition (Figure 8A). To assess the effects of Gel‐EA‐sEVs on myofibroblast transdifferentiation in vivo, the expression levels of α‐SMA and COL‐1 were observed by immunohistochemical and immunofluorescent staining. The results showed the enhanced α‐SMA and COL‐1 expression in the Gel‐EA‐sEVs group compared with other groups, indicating that HDFs in the wounds were transdifferentiated into myofibroblasts (Figure 8B,C,E–H). Additionally, the expression of EGFR was also evaluated with immunohistochemical staining. Consistent with the results in vitro, the expression levels of EGFR in the Gel‐EA‐sEVs and EA‐sEVs groups were notably higher compared with the other groups (Figure 8D,I).

All above results demonstrated that Gel‐EA‐sEVs significantly enhanced diabetic wound healing by promoting re‐epithelialisation and collagen deposition. EGFR expression probably was responsible for the improved functions of skin repair cells.

## 4 Discussion

Functional disorder of HDFs and HEKs caused by hyperglycaemia is one of the main characteristics of diabetic wounds. In the present study, we loaded EA in EVs and proved that EA‐sEVs could enhance the migration and transdifferentiation abilities of HG‐HDFs as well as increase the proliferation and migratory capabilities of HG‐HEKs. Subsequently, we confirmed EGFR was responsible for improved functions of HG‐HDFs and HG‐HEKs by EA‐sEVs. Finally, we constructed Gel‐EA‐sEVs and found that Gel‐EA‐sEVs promoted wound healing by improving re‐epithelialisation, structuring collagen deposition and increasing EGFR expression in diabetic wounds of rodent models.

The typical wound healing processes consist of haemostasis, inflammation, proliferation and remodelling phases. There are various cells in the wound that contribute to wound repair [43]. Dermal fibroblasts, as one of the most important skin repair cells, facilitate the healing process by proliferating and migrating to the wound sites and then differentiating into myofibroblasts to form ECM for reshaping the wound beds [44]. Epidermal keratinocytes, another important skin repair cell, possess the capacity for rapid proliferation and migration during re‐epithelialisation to cover wound surfaces [45]. However, these essential functional behaviours of skin repair cells are dysregulated in hyperglycaemic microenvironments in diabetic wounds, leading to the formation of chronic non‐healing wounds [2, 46]. In our study, HDFs and HEKs were cultured in glucose to simulate a diabetic high‐glucose microenvironment [46, 47] and then were used to evaluate the effects of small‐molecule drugs on improving cellular function.

EA, the most active polyphenol in pomegranates, has shown promising therapeutic potential in disease treatment by improving the functional status of disease‐related cells [8, 48]. As a liposomal small molecule, it is difficult to apply directly for disease treatment because of poor water solubility, low transdermal efficacy and rapid clearance [49]. To overcome the limitations, researchers have developed ingenious delivery systems for EA, including chitosan nanoparticles [50, 51], hydrogels [52] and nanofibers [7]. However, these approaches failed to achieve the efficient intracellular active delivery of EA and faced the challenge of activating the immune system, necessitating the exploration of novel and safe vectors. Extracellular vehicles (EVs) were considered as a next‐generation drug delivery platform due to their excellent biocompatibility, innate cellular communication, higher stability, lower tumorigenicity and ease of engineering [31, 53]. Although the co‐incubation process may not be the most efficient method, it has proven to preserve the integrity and function of sEVs [54]. The loading efficiency of EA inside sEVs was approximately 16.67%. The obtained EA‐sEVs significantly enhanced the migration, transdifferentiation and collagen synthesis abilities of HG‐HDFs, as well as improved the proliferation and migration of HG‐HEKs.

Regarding the mechanism of EA, it has been reported that EA can regulate cellular function by reducing the activation of NF‐κB, downregulating caspase‐3 and promoting the DNA repair process [55]. However, the targets of EA during the improvement of the function of HG‐HDFs and HG‐HEKs are unclear. In our study, by using bioinformatic tools, EGFR, INSR and C‐SRC were predicted as the intersection targets of EA. Adding EA enhanced the expression of EGFR, not INSR and C‐SRC. Erlotinib, as an EGFR inhibitor, effectively blocked the improved function of HG‐HDFs and HG‐HEKs by EA‐sEVs. So, we first reported EGFR as a target of EA for the improvement of cellular functions. EGFR was a well‐known transmembrane glycoprotein and was highly associated with cell proliferation, migration and differentiation [56]. In the field of skin repair, EGFR is vital for maintaining normal skin structure and enhances wound healing through its regulation of re‐epithelialisation, angiogenesis, fibroblast migration and proliferation, as well as inflammation [57, 58]. However, the EGFR expression in diabetic wounds was reported to decrease [59]. Additionally, it was also reported that the HG microenvironment impaired EGFR signalling and attenuated corneal epithelial wound healing [60]. Consistent with these findings, we observed the significant downregulation of EGFR expression in HG‐HDFs and HG‐HEKs, and the treatment with EA‐sEVs elevated its expression. These results suggested that the expression and activation of EGFR were responsible for improved functions of HG‐HDFs and HG‐HEKs by EA‐sEVs.

Although sEVs have been reported as the potential delivery vehicles for wound healing [61, 62], their short half‐life and instability hinder their therapeutic efficacy in clinical application. Loading sEVs in biomaterials [32] may be a powerful solution to sEV instability and realise a controlled sustained release of sEVs in vivo. Silk fibroin scaffolds [32], microneedle arrays [63], microsphere [64, 65], electrospun nanofibers [66, 67] and hydrogels [37, 47, 68] were adopted to achieve sustained and controlled release of sEVs in wound treatment. Especially, GelMA hydrogels have been extensively investigated as a platform to locally deliver sEVs [69]. Generally, the porosity, mechanical properties, swelling behaviour and degradation rate of GelMA hydrogels can be regulated by changing the monomer concentration of GelMA hydrogels and the substitution rate of MA [70]. In our research, three kinds of monomer concentrations of GelMA hydrogels were investigated and the results indicated that GelMA hydrogels with 15% monomer concentration showed lower porosity, higher mechanical strength, lower degradation and swelling ratio. EA‐sEVs were encapsulated in 15% GelMA hydrogels and applied for the treatment of diabetic wounds in rodent models, taking PBS, EA, sEVs, EA‐sEVs and GelMA as controls. Gel‐EA‐sEVs‐treated wounds displayed the fastest closure rate, better re‐epithelialisation, organised collagen deposition and enhanced EGFR expression. Hence, Gel‐EA‐sEVs can be a promising wound dressing material for treating diabetic wounds.

## 5 Conclusions

In conclusion, EA‐sEVs significantly enhanced the proliferation, migration and transdifferentiation of HG‐HDF, as well as the proliferation and migration of HG‐HEKs via activating EGFR. Gel‐EA‐sEVs efficiently facilitated the healing process of diabetic wounds in rodent models by enhancing re‐epithelialisation and collagen synthesis. This study indicated that Gel‐EA‐sEVs can be a promising treatment approach for diabetic wounds by improving the biological functions of HDFs and HEKs.

## Author Contributions

L.T. and Z.W. designed the study and performed most of the experiments. S.C., K.G. and Y.H. performed part of the experiments. L.M., K.M. and J.C. provided technical and material support. Z.W. and S.C. analysed data. L.T. wrote the manuscript. X.L., L.L., X.F. and C.Z. initiated the study and reviewed the manuscript. All of the authors have read and approved the article.

## Ethics Statement

All animal protocols were approved by the Institutional Animal Care and Use Committee of Beijing Sibeifu Laboratory Animal Technology (approval number AWE2021091501).

## Conflicts of Interest

The authors declare no conflicts of interest.

## Supporting information


**Data S1.** Supporting Information.

## Data Availability

The data that support the findings of this study are available from the corresponding author upon reasonable request.
