# Supplementary material for: Ellagic Acid‐Loaded sEVs Encapsulated in GelMA Hydrogel Accelerate Diabetic Wound Healing by Activating EGFR on Skin Repair Cells
Source: Cell Prolif. 2025 May 19;58(10):e70064. doi: 10.1111/cpr.70064 (PMC12508689; doi:10.1111/cpr.70064)
Supplement: Supplementary file 1 — Data S1. Supporting Information. [file CPR-58-e70064-s001.docx]

Supplementary Materials for

**Ellagic acid-loaded sEVs encapsulated in GelMA hydrogel accelerate diabetic wound healing by activating EGFR on skin repair cells**

Lige Tian^1,2,†^, Zihao Wang^2,3,†^, Shengqiu Chen^2,4,†^, Kailu Guo^1,2^, Yaying Hao^2,5^, Liqian Ma^2,5^, Kui Ma^2,5^, Junli Chen^2,5^, Xi Liu^2,5*^, Linlin Li^6,*^, Xiaobing Fu^2,5,*^, and Cuiping Zhang^2,5,*^

^1^ College of Graduate, Tianjin Medical University, Tianjin, 300070, China;

^2^ Medical Innovation Research Department, PLA General Hospital, Beijing, 100853, China;

^3^ Chinese PLA Medical School, Beijing, 100853, China;

^4^ Innovation Research Center for Diabetic Foot, West China Hospital, Sichuan University, Chengdu 610041, China;

^5^ PLA Key Laboratory of Tissue Repair and Regenerative Medicine, Beijing, 100048, China.

^6^ Beijing Key Laboratory of Micro-Nano Energy and Sensor, Center for High-Entropy Energy and Systems, Beijing Institute of Nanoenergy and Nanosystems, Chinese Academy of Sciences, Beijing 101400, China

* Corresponding author.

E-mail addresses: liuxipla@163.com (X. Liu), lilinlin@binn.cas.cn (L. Li), fuxiaobing@vip.sina.com (X. Fu), zcp666666@sohu.com (C. Zhang).

^†^ These authors contributed equally to this work.

**This Supplementary Materials includes:**

Figures. S1 to S2

Tables S1 to S2


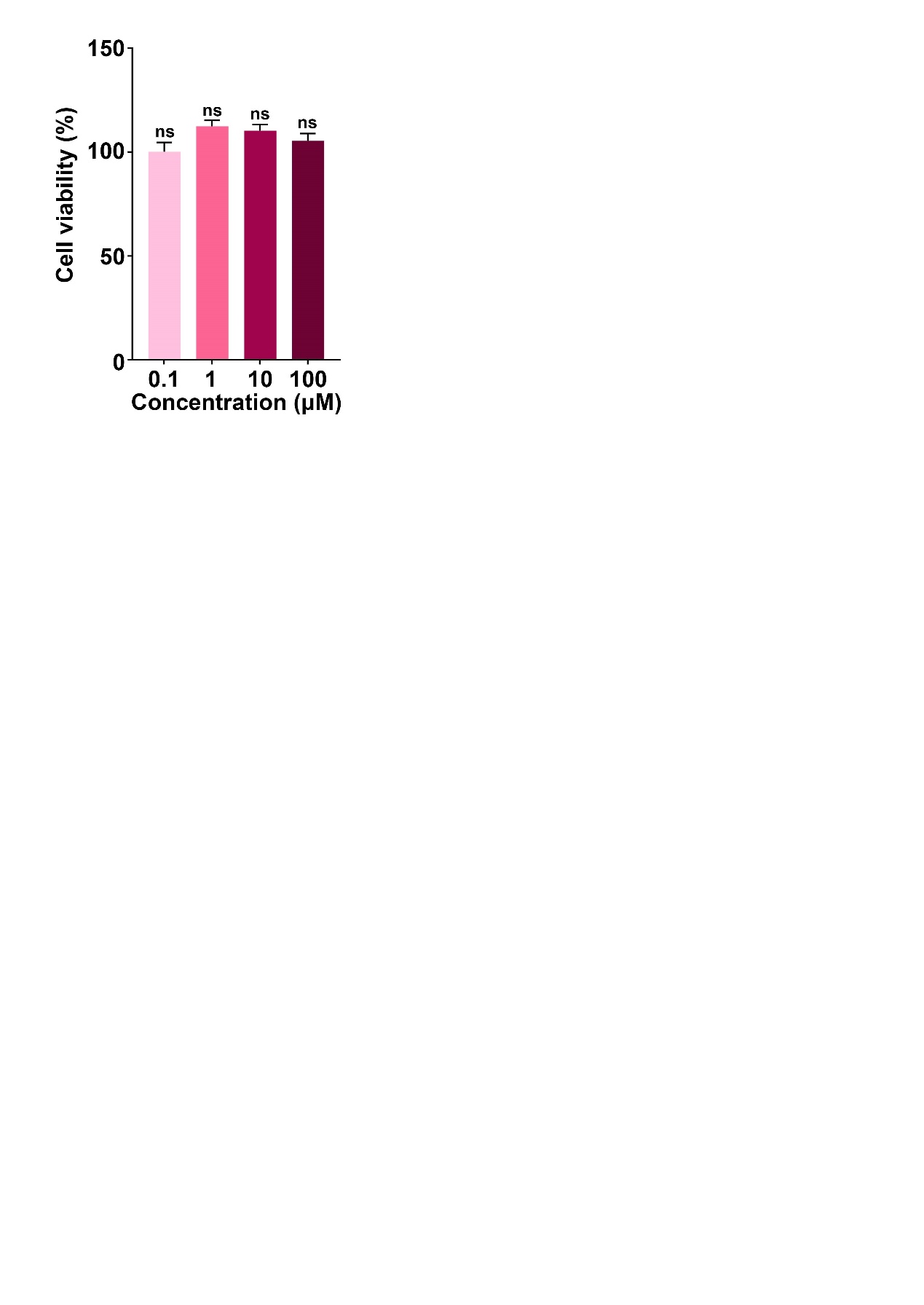


Figure S1 Effects of EA with different concentrations on the cell viability of HG-HDFs. The data are presented as the mean ± SD. Differences among the groups were examined with one-way ANOVA with Tukey’s posttest. *ns*, not significant.


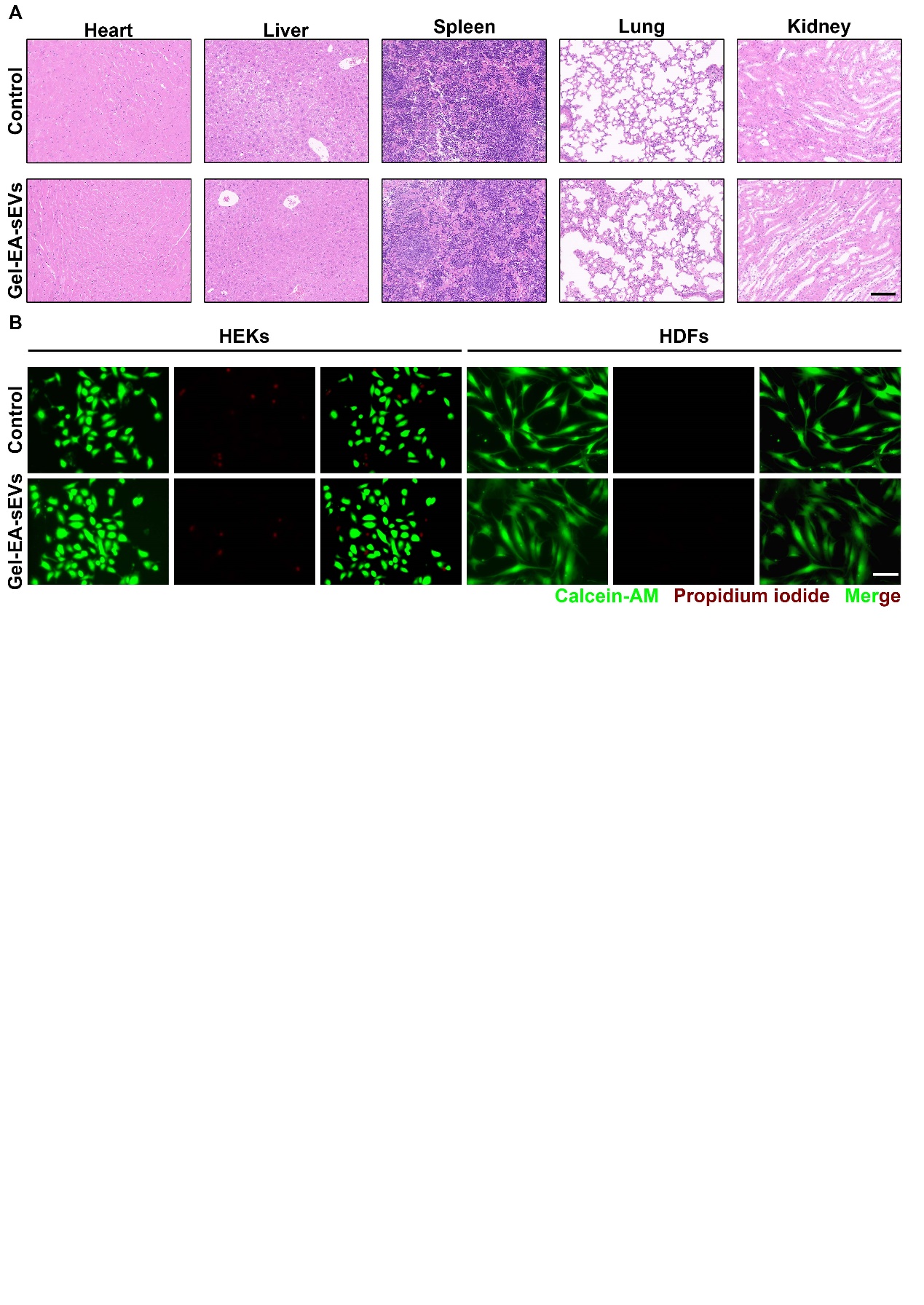


Figure S2 Biocompatibility evaluation of Gel-EA-sEVs. (A) The H&E staining images of heart, liver, spleen, lung, and kidney in mice with wounds treated with Gel-EA-sEVs on day 14 after surgery; scale bar, 100 μm. (B) Representative live/dead staining images of HG-HEKs and HG-HDFs; scale bar, 50 μm.


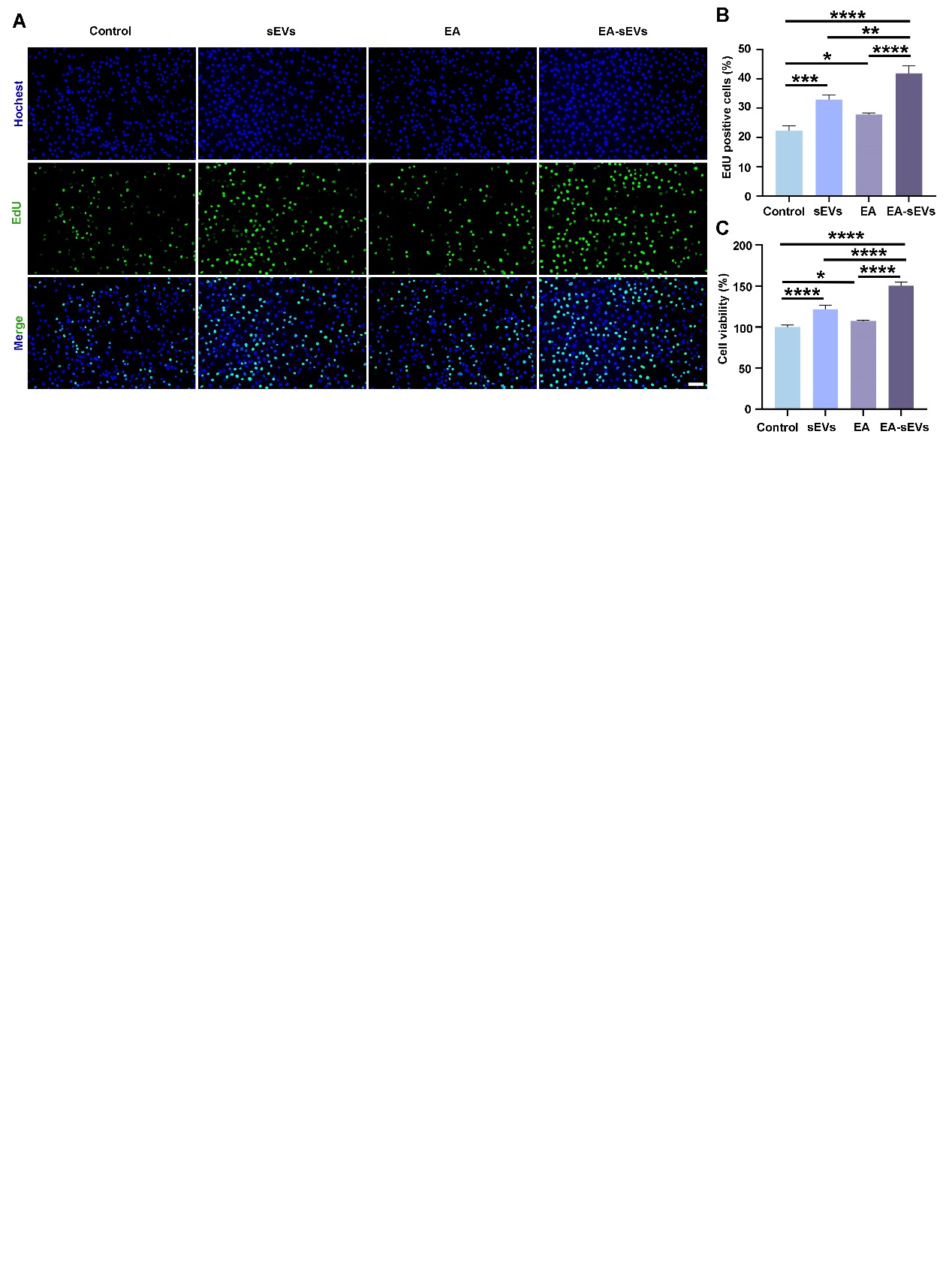


Figure S3 EA-EVs promoted the proliferation of HG-HDFs. (A) EdU immunofluorescent staining images of HG-HDFs treated with different groups; scale bar, 100 μm. (B) Quantitative analysis of (A) (n = 3). (C) The CCK8 results of HG-HDFs treated with different groups (n = 6). The data are presented as the mean ± SD. Differences among the groups were examined with one-way ANOVA with Tukey’s posttest. *p < 0.05, **p < 0.01, ***p < 0.001, ****p < 0.0001.


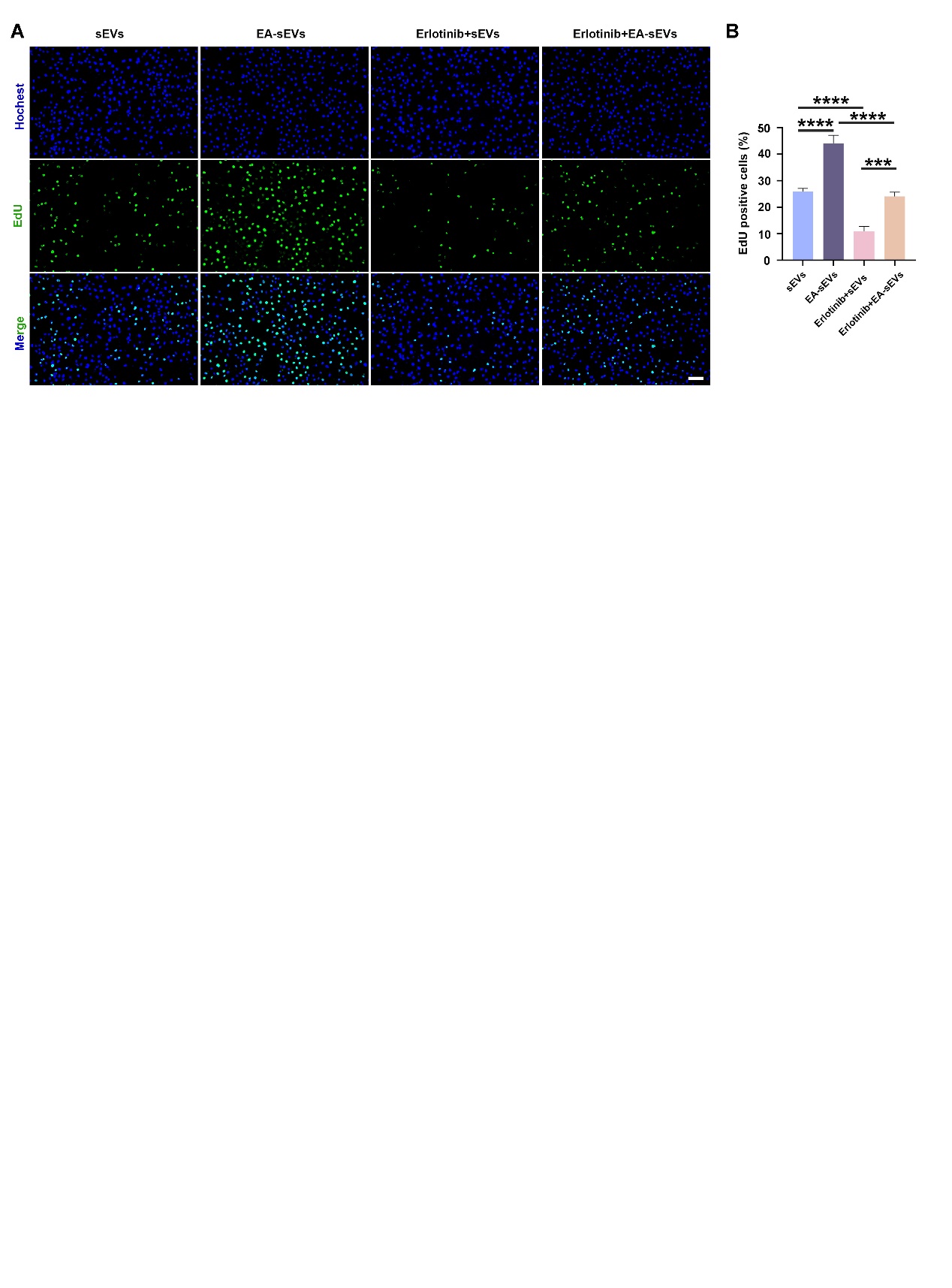


Figure S4 Erlotinib blocked the improved proliferation capability of HG-HDFs by EA-sEVs. (A) EdU immunofluorescent staining images of HG-HDFs treated with different groups; scale bar, 100 μm. (B) Statistical analysis of (A) (n = 3). The data are presented as the mean ± SD. Differences among the groups were examined with one-way ANOVA with Tukey’s posttest. ***p < 0.001, ****p < 0.0001, ns, not significant.


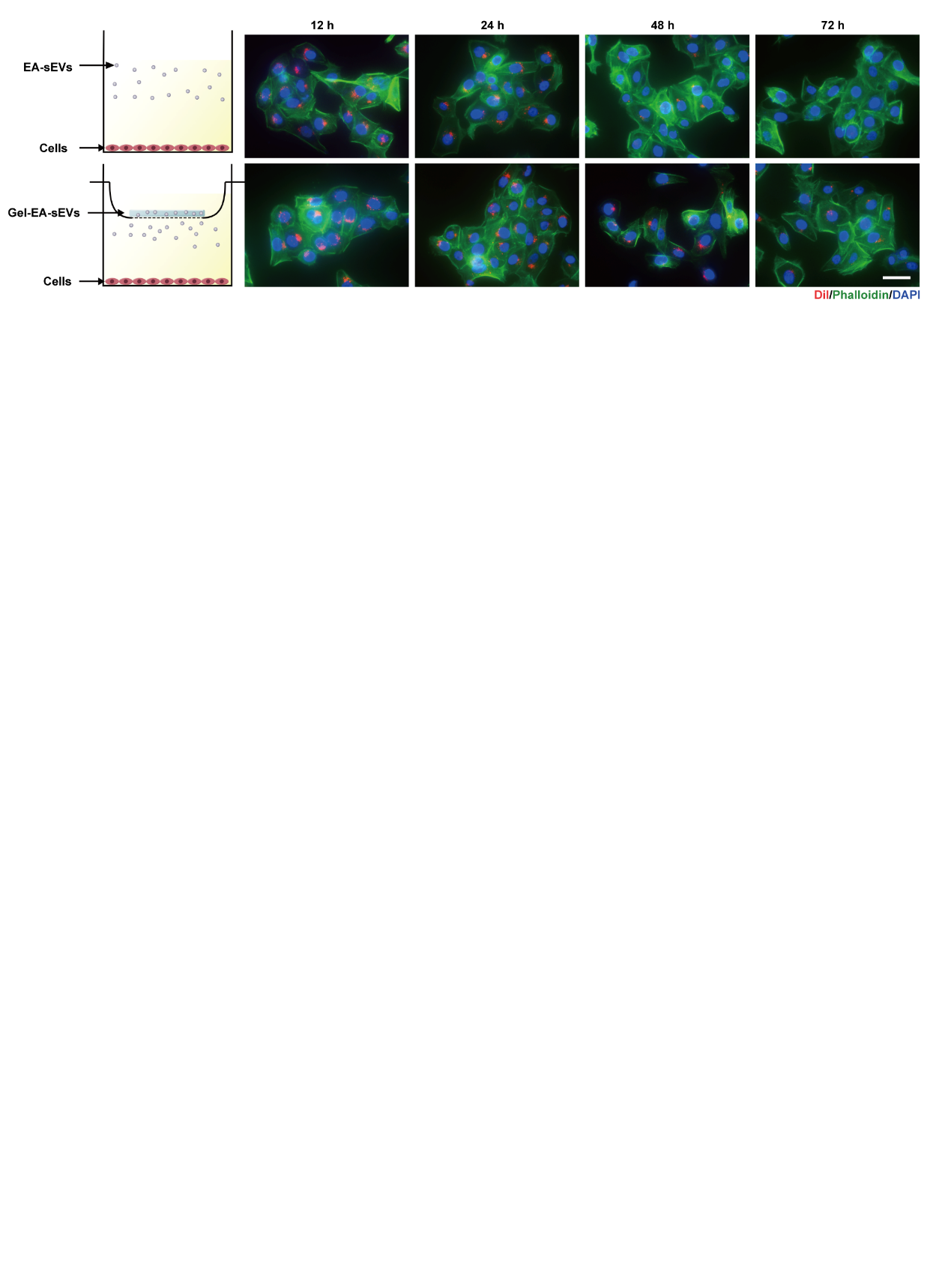


Figure S5 Illustration and fluorescent images reflecting the retention time of EA-sEVs in free form or encapsulation form inside HG-HEKs; scale bar, 50 μm. Blue: DAPI, red: Dil, green: phalloidine.

**Tables S1 to S2**

**Table S1** **The solvent program of HPLC.**

| Time (minutes) | ACN% | H_2_O (0.05%TFA) % |
| --- | --- | --- |
| 0.0 | 10 | 90 |
| 8.0 | 90 | 10 |
| 13.0 | 90 | 10 |
| 13.1 | 10 | 90 |
| 16 | 10 | 90 |

**Table S2 Sequences of specific primers used for qPCR analysis**

| **Gene name** | **Primer sequence (5′ to 3′)** |
| --- | --- |
| GAPDH | F: GTCCCAGCTTAGGTTCATAG  R: GATGGCAACAATCTCCACTTTG |
| COL1A1 | F: GAGGGCCAAGACGAAGACATC  R: CAGATCACGTCATCGCACAAC |
| FN1 | F: CGGTGGCTGTCAGTCAAAG  R: AAACCTCGGCTTCCTCCATAA |
| ACTA2 | F: AAAAGACAGCTACGTGGGTGA  R: GCCATGTTCTATCGGGTACTTC |
